# A Rare Bullet Trajectory: A Case Report of an Abnormal Gunshot Wound

**DOI:** 10.7759/cureus.88096

**Published:** 2025-07-16

**Authors:** Sami Ataman, Mustafa K Ayranci

**Affiliations:** 1 Emergency Department, Muradiye State Hospital, Van, TUR; 2 Emergency Department, Necmettin Erbakan University Hospital, Konya, TUR

**Keywords:** abnormal bullet trajectory, gunshot wound, multidisciplinary approach, penetrating trauma, traumatic injury management

## Abstract

Gunshot wounds (GSWs) to the thoracic region are often associated with high mortality due to the injury of vital organs such as the heart, lungs, and aorta. Abnormal bullet trajectories complicate diagnosis and treatment, particularly when the bullet remains lodged within the body and no exit wound is present. This case report describes a rare instance of a gunshot wound entering through the anterior chest wall and ultimately lodging in the rectal region without an exit wound. Initial chest and abdominal imaging showed no acute pathology; however, advanced imaging, including contrast-enhanced abdominal and chest computed tomography (CT) scans, revealed that the bullet followed an atypical trajectory, traversing the small bowel, bladder, and pubic ramus before settling in the inferior ischioanal region. The patient underwent successful surgical intervention for both bowel and bladder injuries. This case highlights the importance of comprehensive secondary assessments, including advanced imaging, in identifying abnormal bullet trajectories and guiding appropriate management.

## Introduction

Gunshot wounds (GSWs) to the chest are generally associated with high mortality due to the presence of vital organs such as the heart, lungs, and aorta in the thoracic region [1]. Common emergency conditions include hemothorax, pneumothorax, cardiac injuries, and hemorrhagic shock. Typically, gunshot wounds present with both an entrance and exit wound. However, in some cases, particularly involving low-caliber or low-velocity firearms, bullets may remain within the body without creating an exit wound [2-5]. Thus, in such cases, the key anomaly is not the absence of an exit wound per se, but the unusual internal trajectory that bypasses vital thoracic structures and lodges in a distant anatomical region, such as the pelvis. Typically, a gunshot wound entering through the thoracic region would be expected to injure vital thoracic organs such as the lungs, heart, or major vessels. However, in the present case, the bullet bypassed these structures and caused injuries to abdominal and pelvic organs, representing an atypical clinical trajectory that deviates from anatomical expectations. To the best of our knowledge, there have been no prior reports describing a gunshot wound entering through the anterior chest wall and lodging in the rectal region without an exit wound. The purpose of this case report is to present this anomalous bullet trajectory. This case also highlights the clinical importance of thoroughly evaluating and imaging other body regions, even when initial findings in the apparent zone of injury are unremarkable, to avoid missing atypical internal trajectories.

## Case presentation

**Figure 1:**
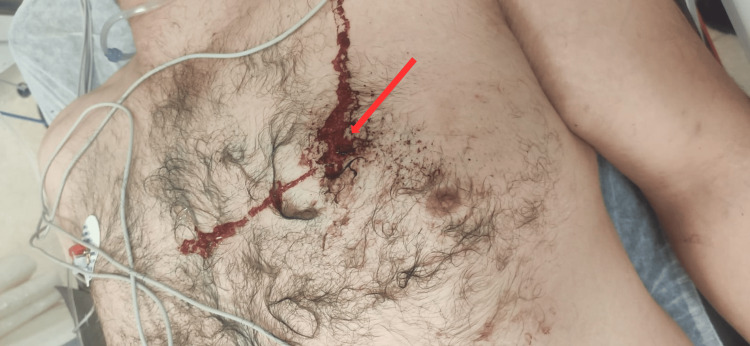
Gunshot entry wound Entry wound on the anterior chest at the level of the third intercostal space (arrow).

A 30-year-old male patient presented to the emergency department on his own, seated and conscious, reporting a gunshot wound to the left side of the chest in the upper thoracic region (Figure 1).

He was evaluated as a level 2 triage case due to penetrating thoracic trauma with abdominal symptoms. He also complained of diffuse abdominal pain. On initial evaluation, the patient's vital signs were stable, and he was alert with a normal neurological examination. Physical examination revealed a 0.5 cm bullet entry wound located at the level of the third intercostal space on the anterior left chest. No exit wound was identified. Respiratory examination was unremarkable, with no rales, rhonchi, or abnormal breath sounds. Abdominal examination revealed tenderness across all quadrants without signs of peritoneal irritation, such as rebound or guarding. A urinary catheter was inserted and drained 250 cc of hemorrhagic fluid.

Chest X-ray did not reveal any acute pathology. Emergency bedside ultrasound showed no significant free fluid in the abdominal cavity. The balloon of the urinary catheter was visualized within the bladder lumen, and heterogeneous echogenicity suggested a hematoma. No pleural effusion was observed in the left pleural space. Transthoracic echocardiography performed by a cardiologist revealed no major valvular pathology, although a minimal pericardial effusion was noted anterior to the right ventricle, without signs of tamponade or elevated intrapericardial pressure.

**Figure 2:**
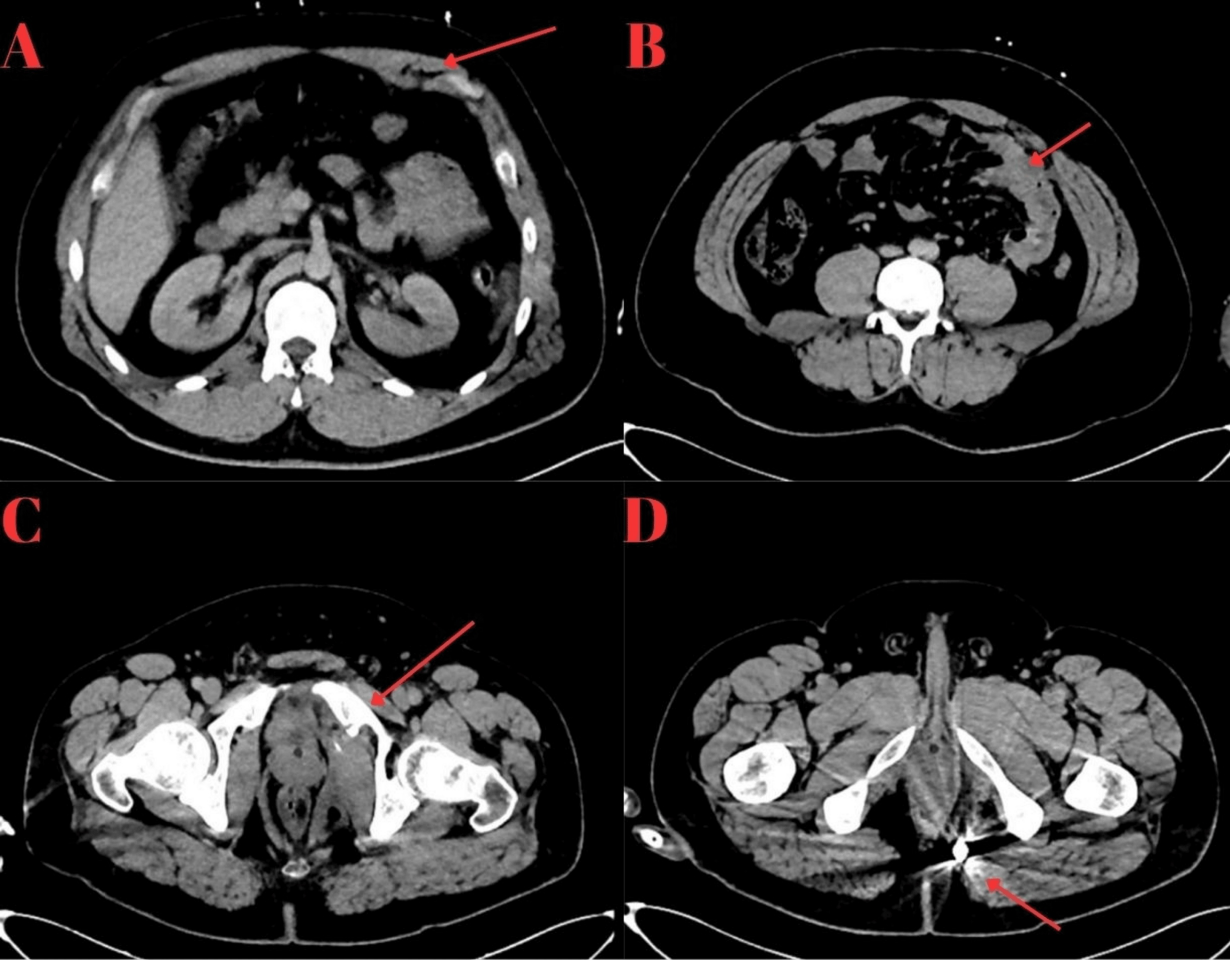
Contrast-enhanced CT scan images of the abdomen and pelvis (A) The arrow indicates free air in the anterior abdominal wall. (B) The arrow shows a suspected area of intraperitoneal hemorrhage adjacent to the bladder. (C) The arrow points to a fracture line in the left pubic ramus. (D) The arrow identifies a metallic foreign body lodged in the left gluteal region. CT: computed tomography

A contrast-enhanced chest computed tomography (CT) scan demonstrated no evidence of acute parenchymal trauma, hemopneumothorax, or rib fractures. However, the abdominal CT scan (Figure 2) revealed significant free air in the anterior abdominal wall and the left lower quadrant, along with subcutaneous air density in the left upper quadrant. Density changes in the left lateral bladder suggested intraperitoneal hemorrhage. A fracture line was identified in the left pubic ramus. A metallic foreign body was visualized in the left gluteal region. Based on the location and trajectory of the bullet, it was hypothesized that the projectile had traveled from the left upper quadrant through the small bowel loops, injuring the left lateral bladder wall and left pubic ramus before lodging in the ischioanal fossa.

After radiological confirmation of abdominal organ injury, the patient was urgently taken to the operating room by the general surgery and urology teams for exploratory laparotomy. Intraoperative findings included multiple perforations in the transverse colon, sigmoid colon, and small intestine, with nine separate perforations spanning approximately 120 cm of the small bowel. Full-thickness perforations were also observed in both the transverse and sigmoid colon. The affected segments of the bowel were resected, and a loop ileostomy was created. During the operation, the urology team identified a 1 cm perforation in the anterior bladder wall, which was repaired primarily. Following surgery, the patient was admitted to the surgical ward for postoperative monitoring and recovery.

## Discussion

Abnormal bullet trajectories are critical for accurately identifying injured organs and planning appropriate surgical interventions. The thoracic cavity contains vital organs, including the heart, lungs, and aorta, rendering thoracic injuries particularly severe. Common diagnoses, such as hemopneumothorax and cardiac tamponade, are usually the primary focus; consequently, initial assessments may neglect other potential injuries. Bullets do not simply travel in a linear trajectory but often exhibit rotational motion, which can cause extensive damage to surrounding tissues [6]. The study by Ro et al. demonstrates how bullets can follow unpredictable, non-linear trajectories within soft tissues, although the entry site in the case involved the knee, highlighting the diagnostic value of computed tomography in understanding such anomalies [7]. Furthermore, impact with hard tissues such as bones can result in fractures and alter the bullet's trajectory. If the bullet's kinetic energy is not fully dissipated upon impact, it may deviate from its initial path. Several cases of gunshot wounds with abnormal bullet trajectories have been documented in the literature [3-5,8]. Similarly, Wankhade et al. reported a case in which the bullet was initially untraceable despite clinical evidence of a gunshot wound, underlining how misleading superficial findings can be in firearm injuries [9]. In such cases, additional radiological evaluations are essential for accurate diagnosis.

Bullets fired at close range possess higher kinetic energy, making the detection of their trajectories more challenging. Both entrance and exit wounds must be carefully evaluated, and the possibility of a retained bullet should always be considered. In our case, no exit wound was identified, necessitating the use of advanced imaging techniques. Furthermore, the patient's abdominal pain and hematuria suggested that the bullet had passed through the abdominal cavity. Bedside ultrasound and echocardiography were performed to evaluate vital organs, including the heart and lungs, revealing no acute pathology. These findings suggested that the bullet may have followed an atypical subcutaneous trajectory, a rare and diagnostically challenging path that could be easily overlooked during initial evaluations. Subsequent CT imaging revealed free air in the abdominal cavity, bladder injury, and a fracture of the pubic ramus. Based on these findings, we inferred that the bullet traversed abdominal organs, struck the bladder and pubic ramus, altered its trajectory, and ultimately lodged in the ischioanal region. Similar diagnostically challenging bullet trajectories have been reported in the literature, highlighting the need for high clinical suspicion and thorough imaging in such cases [10].

## Conclusions

This case underscores the critical role of comprehensive secondary assessments and the value of multidisciplinary management in gunshot wounds, especially those with atypical trajectories that may evade initial detection. Furthermore, it highlights that thoracic entry wounds should not lead clinicians to prematurely rule out intra-abdominal or pelvic injuries, particularly when initial thoracic imaging is unremarkable.
